# Satellite DNA Genomics: The Ongoing Story

**DOI:** 10.3390/ijms262311291

**Published:** 2025-11-22

**Authors:** Manuel A. Garrido-Ramos, Miroslav Plohl, Eva Šatović-Vukšić

**Affiliations:** 1 Departamento de Genética, Facultad de Ciencias, Universidad de Granada, 18071 Granada, Spain; mgarrido@ugr.es; 2 Division of Molecular Biology, Ruđer Bošković Institute, 10000 Zagreb, Croatia; plohl@irb.hr

**Keywords:** repetitive DNA, satellite DNA, satellite DNA library, repetitive DNA library, transposable elements, satellite DNA genomics, genome biology, genome evolution, methodological approaches in satellite DNA research

## Abstract

Tandemly repeated non-coding sequences, widely known as satellite DNAs (satDNAs), are extremely diverse and highly variable components of eukaryotic genomes. In recent years, advances in high-throughput sequencing and new bioinformatics platforms have enabled in-depth studies of all (or nearly all) tandem repeats in any genome (the satellitome), while a growing number of telomere-to-telomere assemblies facilitates their detailed mapping. Research performed on a large number of non-model plant and animal species changed significantly the “classical” view on these sequences, both in an organizational and functional sense, from ballast compacted in the form of heterochromatin to elements that are important for structuring the entire genome, as well as for its functions and evolution. The diversity of repeat families, and the complexity of their intraspecies and interspecies distribution patterns, posed new questions, urging for species-by-species comparative analyses. Here we integrate some basic features of different forms of sequences repeated in tandem and rapidly growing data evidencing extensive dispersal of satDNA sequences in euchromatin, their putative roles and evolutionary significance. Importantly, we also present and discuss various issues brought on by the use of new methodological approaches and point out potential threats to the analysis of satDNAs and satellitomes.

## 1. Introduction

Genomic fraction composed of tandem repeats was first discovered as a “satellite” band separated from the bulk of genomic DNA in gradient-density centrifugation experiments [1,2,3], hence the name satellite DNA (satDNA). SatDNAs were traditionally described as highly abundant non-coding DNA sequences repeated in tandem that form long arrays clustered on chromosomal locations such as heterochromatin and centromeric regions [4,5,6,7]. The updated definition to this concept will be discussed below. SatDNAs and interspersed repetitive sequences, transposable elements (TEs), build the largest part of eukaryotic genomes [8,9].

High-throughput methodologies and increased research interest enabled rapid accumulation of satDNA data on the genomic scale in a wide variety of species and promoted significant advances in understanding diverse aspects of satDNA genomics and functionality. SatDNAs are now perceived as important genome organizers, and can be linked with genome/chromosomal architecture, function, evolution and speciation, gene expression regulation, stress response, adaptations and diseases [10,11,12,13,14,15,16,17,18,19].

Genomic studies revealed surprisingly high diversity of satDNAs in sequence, abundance, organizational patterns and the outcomes of evolutionary processes [10,14,20,21,22,23,24,25,26,27,28,29,30,31,32,33,34,35,36]. They establish a genome-wide network of considerably greater complexity than the conventional notion of “long arrays confined to heterochromatin”, a comprehension which has significantly changed and reshaped our understanding of satDNAs [10]. It is now evident that almost every species or species group represents a model for some specific aspect of satDNA genomics (for example, TE-linked satDNAs in oysters [26], and satDNAs in karyotype diversity, genome expansion and speciation in wood white butterflies, [21]).

Diversities in almost every aspect urge for more comparative studies in different organisms to better understand causes, consequences and functional constraints that may profile satDNAs and their landscapes. In this review we focus on recent advancements in satDNA genomics, and on new data needed to broaden the traditional view on satDNAs, by taking into consideration all forms of tandem repeats that can be found within the genome. And in order to discuss or redefine what satDNA is, as we will do throughout and at the end of this article, it is necessary to refer first to its different characteristics which are the object of the following sections. We also want to draw attention to some important questions posed by using current high-throughput methodologies, emphasizing risks in satellitome research and its potential outcomes, and encouraging further studies on related groups of non-model species.

## 2. Advanced Methodologies in SatDNA Research

The early methodological approaches included restriction enzyme digestion of genomic DNA followed by electrophoretic detection of the characteristic ladder-like pattern of repeat units [5]. Cloning of these fragments enabled the sequencing of a number of monomers, determination of consensus sequences, and preparation of fluorescence in situ hybridization (FISH) probes for chromosomal mapping.

At the genomic scale, the high sequence similarity among repeat units represents a major obstacle to the sequencing and accurate assembly of repetitive DNAs. Consequently, satDNAs, and repetitive sequences in general, have been referred to as the ”genomic dark matter”, as they were not only poorly represented in the genome assemblies generated by Sanger and later sequencing technologies, but had also long remained enigmatic regarding their biological roles [37,38,39,40,41].

Important steps forward in satDNA research were achieved by new methodologies, next-generation sequencing (NGS), and third generation long-read DNA sequencing [39,42,43,44]. The strategy using NGS short-read low-coverage (<0.5×) genome sequencing and specialized bioinformatics pipelines (such as widely used RepeatExplorer and TAREAN) make possible the detection of all, or nearly all, repetitive DNAs in any species without the need for genome assembly [45,46,47,48,49,50,51,52]. These methods allow the identification of the inventory of repetitive DNAs in a genome, the repeatome [53] or of satDNAs, the satellitome [50]. Nevertheless, listing the satDNAs cannot explain their localization and sequential order, and the NGS approach must be combined with in situ (FISH) and/or in silico mapping. Advantages and disadvantages as well as the importance of combining these methods will be attended later.

With the help of appropriate bioinformatics tools, long reads obtained by the third-generation sequencing can significantly improve the precision of the telomere-to-telomere chromosome assembly, including the regions occupied by long arrays of satDNAs [39,42,54]. Application of this methodology is progressing rapidly, adding more organisms and introducing complex comparative studies of precisely annotated repetitive sequences [38,55,56]. For example, repetitive sequences were precisely annotated in the recent study of six ape species, enabling comparisons of primate-specific centromeric alpha-satDNA arrays, and reconstruction of their evolution [57]. However, difficulties in assemblies of tandem repeats are still not fully eliminated, due to the chromosomal distribution of highly similar satDNA monomers. For example, NGS-detected major pericentromeric satDNA (26.5% of the genome) of the beetle *Tenebrio molitor* is significantly underrepresented in the genomic scaffolds assembled from long-reads, constituting only 0.4% of the assembly [58]. Similarly, satDNA of the ladybird *Adalia bipunctata* located in the pericentromeric heterochromatin builds 9.98% of the genome according to the NGS reads but is about 3 times less represented in the chromosome-level genome assembly [25].

## 3. Distinction According to the Repeat Unit Length

Based on monomer length, tandemly repeated genomic sequences have traditionally been classified into satDNAs, minisatellites, microsatellites and telomeric repeats. Microsatellites typically consist of 2–10 base pairs (bp) repeat units, minisatellites of 11–60 bp and satDNAs of repeats longer than 60 bp [10,59,60]. Mini- and microsatellites are mainly found in euchromatin while telomeric repeats at chromosome ends. SatDNAs with monomers > 500 bp are sometimes called macrosatellites, to emphasize their large monomer size and a possible composite structure [11]. However, the above classification is only approximate. For example, long arrays of tandem repeats composed of 5 bp monomers build heterochromatin of *Drosophila*, particularly on the Y chromosome, such as the most abundant (AAGAG)_n_ satDNA [61]. Human satDNAs II and III are also composed of 5 bp long motifs and located in the pericentromeric regions of particular chromosomes [62].

As it will be discussed below, novel approaches have revealed the broad presence of all forms of tandem repeats, composed either of long or of short repeat units, in both heterochromatin and euchromatin, erasing the already loose boundaries of traditional divisions according to the repeat unit length, array length and heterochromatin/euchromatin localization [10,14,59,60]. In agreement with the suggestion by Ruiz-Ruano et al. [50], the traditional definition of satDNAs could therefore be extended onto all forms of non-coding tandem repeats appearing in the genome. Nowadays, despite this, the terms micro- and minisatellites, differentiated from satDNA, continue to be used for practical reasons.

Microsatellites are often called simple sequence repeats or short tandem repeats (STRs). They can be found literally everywhere in the genome, including within or next to other repetitive elements, and can be distributed in uneven and nonrandom manner [63]. For example, they are a constitutive part of TEs of the Helitron superfamily [64]. Nevertheless, microsatellites are mostly found as standalone sequences in up to ~100 bp long arrays. In humans, STRs of 1–6 bp motifs build a significant amount 6.77% of the genome, annotated on 1.5 million dispersed loci [65]. The loci are highly variable in number of repeats because of slipped mispairing and unequal crossover. Variability of STRs in the vicinity or within genes normally does not affect gene function, unless the arrays expand above some threshold value. Expanded STR arrays have been identified as a specific cause of many diseases, with the severity of symptoms increasing with the array length. Interestingly, disorders are neurological or neuromuscular (such as Huntington disease, myotonic dystrophy, fragile X and frontotemporal dementia), raising the hypothesis that STRs participated in the evolution of the human brain [65,66,67]. Among other organisms, repeat copy number alterations affect behavior of prairie voles [68] or phenotype in plants [69]. There is an emerging role of microsatellites as enhancer regulatory elements. For example, in Ewing sarcoma, hundreds of GGAA repeats can be converted into active enhancers when bound by an EWS-FLI1 fusion protein [70,71]. Microsatellites may play a major role in transcriptional regulation, with multiple transcription factors binding to their sequences and exerting complex, and sometimes unforeseen, effects on transcriptional activity [72,73,74,75,76]. Nowadays, machine learning approaches aid the identification of such roles of microsatellites [77], while bioinformatics tools assist the correct profiling of these regions [78].

STRs clustered at chromosome ends form telomeres, structures that protect genomic DNA from degradation and prevent chromosome fusions. The most common among Metazoans and plants are (TTAGGG)_n_ and (TTTAGGG)_n_, respectively, and derivatives of these motifs. The arrays on chromosome ends are variable in length, and subject to shortening in cell divisions upon stress and aging, while the length is maintained in reproductive cells by specific mechanisms [79]. Telomeric repeats can be found as interstitial genomic sequences, where they compose unstable, highly polymorphic arrays associated with protein components similar to those involved in telomere maintenance, affecting genome stability by initializing chromosomal rearrangements [79,80].

Minisatellites usually build short arrays of few hundreds bp dispersed in euchromatin, but are also clustered in heterochromatin [6,10]. Minisatellite loci are characterized by the high variability in the number of repeats in arrays. Minisatellites can be a dominant component of the satellitome, such as in the freshwater crab *Pontastacus leptodactylus* where they compose 240 out of 258 sequences repeated in tandem [81].

Individual differences in short arrays can be easily evaluated in loci occupied by microsatellites and minisatellites, known also as the variable number of tandem repeats (VNTRs), and applied as markers in medicine and in the identification of individuals and populations [6,82,83].

## 4. SatDNA Sequences and Sequence Changes

Satellite DNAs display diverse origins from various genomic sources and follow different evolutionary pathways and processes, as will be discussed in this section. The parallel existence of many unrelated satDNAs within a genome supports the idea that tandem repeats can originate from virtually any DNA sequence, ranging from just a few bp to over 1 kb [6,8,9,84]. A model explaining satDNA formation proposes that short tandem arrays, initially dispersed throughout the genome, can undergo successive stages of birth, dissemination and clustering [50]. This model, consistent with the “library hypothesis” ([85]; see below), suggests that these scattered short arrays may amplify at specific loci, forming long arrays, while others remain short. Thus, satDNA would arise in tandem duplication yielding at least two monomers and their subsequent amplification by mechanisms of non-reciprocal exchange, which promote the formation of longer arrays [20]. In contrast to the homogeneity observed in long arrays, short arrays often exhibit greater heterogeneity, suggesting a relationship between sequence homogenization and array length [20,86].

### 4.1. SatDNA Sources

Notably, a significant proportion of the sequences from which satDNAs originate is derived from other repetitive sequences, including TEs and different forms of sequences repeated in tandem. Concerning TEs, they play an important role in the evolution of satDNA by the tandemization of parts of their structural components, which in some instances, expand into arrays that give rise to new satDNAs. SatDNAs can derive from DNA transposons, including “cut-and-paste” transposons and Helitrons, as well as MITEs, and from Penelope elements, SINEs, LINEs and LTR retrotransposons (for example [14,22,23,24,26,32,33,87,88,89,90,91,92,93,94,95,96]). A detailed overview of the various mechanisms of satDNA formation and of the relationships between TEs and satDNAs is presented in [19]. Additionally, hypothesis that explains the existence of particular types of monomers, namely composite satDNA monomers which display multiple subsequent stretches of similarity to various TEs, was recently given by Šatović-Vukšić et al. [19]. Euchromatic regions of different species are regularly replete with short arrays of tandem repeats associated with TEs that can represent important sources of tandem repeats and vehicles in their dispersal. Of particular interest are DNA transposons of the Helitron superfamily, which often incorporate short arrays of conventional satDNA monomers that can expand into standalone satDNA arrays [19,26,64,94,96,97,98,99,100,101]. Noteworthy is the presence of short tandem repeat arrays embedded within the 3′ untranslated regions (UTRs) of LTR retrotransposons, which have served as an important source of novel satDNAs in plants [102,103,104]. In this manner, TEs and transposition promote satDNA dissemination, as the dispersal of TE-derived and TE-embedded repeat arrays throughout the chromosomes of a given species can provide multiple “seeds” for the formation of future long arrays of satDNA.

Concerning tandem repetitive sequences, the intergenic spacers of the ribosomal RNA (rRNA) genes [105,106,107,108,109,110,111,112,113,114,115] have been identified as sources of new satDNAs. And both microsatellites [116] and minisatellites [27] have been identified as precursors of new longer repeat unit satDNAs. Evidence also suggests that certain satDNAs can evolve from pre-existing ones, forming groups of related satDNAs [20,22,27,117,118,119] and that there are satDNAs whose current repetitive unit evolved from units with shorter lengths through cycles of duplication and divergence [22,27,116,120,121,122].

### 4.2. Structural Features

Monomers of certain satDNAs possess distinctive structural features—such as intrinsically bent DNA helices, inverted repeats, palindromes, or conserved sequence motifs—that may function as important structural or regulatory signals, particularly within centromeric regions [11,123,124,125]. In two mouse species, *Mus musculus* and *M. spretus*, packaging of divergent satDNAs is determined by a sequence-dependent DNA shape needed for interaction with conserved protein components in centromeric and pericentromeric areas [126]. Such structural features may, in turn, facilitate long-term persistence of some satDNAs [124]. It could be, therefore, proposed that, depending on a putative functional role and the location in the genome, tandem repeats may be either propagated and preserved through long evolutionary periods or lost. In addition, it has been proposed that structural features may provide signals that aid the mechanisms responsible for the fast proliferation of satDNA repeats, both within arrays and throughout the genome [127]. Tandemization of DNA segments could be aided by direct, inverted or palindromic motifs and by employing various mechanisms (reviewed in [19]).

### 4.3. Sequence Variability

Sequence variability among repeat units is often low within the arrays of a satDNA, usually up to about 10%. It has been proposed that array homogeneity is maintained by the non-independent evolution of satDNA monomers, concerted evolution [128,129,130]. According to this view, mutations are homogenized (spread or eliminated) within a family of repeat units in the array and across arrays in the genome in a process driven by mechanisms of non-reciprocal DNA exchange (such as unequal crossover, rolling-circle amplification or processes related to transposition), which are involved in the intragenomic maintenance of satDNA arrays and are fixed in a population (species) by sexual reproduction. The sequence variability profile is therefore the result of a balance between two opposing processes, the mutation rate and the rate of homogenization among monomers [10,84,128,130,131,132,133,134]. Processes of sequence homogenization are less efficient at array ends than in the central parts, generating there more divergent variants that can be subsequently amplified into novel satDNA families [127,131,135,136,137]. Thus, homogenized mutations (often indicated as diagnostic mutations) in orthologous satDNAs would be species distinctive and follow phylogeny [138]. Alternatively, homogeneity of satDNA arrays can be the consequence of recently amplified monomer sequences. Amplification is followed by an accumulation of mutations, the gradual decay of monomers, and their replacement by a new homogeneous array built by the amplification of some monomer variant formed in the process of decay or of some unrelated sequence [139]. High-throughput analyses of the satellitomes of two grasshopper species enabled the raising of a model suggested by Camacho et al. [20], of the recursive cycles of amplification followed by the accumulation of variability by mutations and the subsequent eventual degeneration, leading to evolutionary pathways of satDNAs that are largely contingent. Thus, amplification would be the intragenomic homogenizing and the intergenomic diversifying force of satDNA, so concerted evolution promptly emerges after lineages split, while non-concerted evolution can persist for long in the reduced or absence of homogenizing events [20]. Reduced homogenization and exceptions from concerted evolution can be because of homology of satDNAs with TEs, satDNA location and organization, evolutionary and biological factors or functional constraints [20,86,121,122,140,141,142,143,144,145,146,147,148,149,150]. A combination of both evolutionary scenarios, concerted and non-concerted evolution, can be envisaged [149]. According to the model of Camacho et al. [20], amplification–degeneration cycles enable satDNA families to avoid extinction by reducing sequence divergence through periodic amplification of a variant; although, eventual degeneration occurs once sequences diverge substantially from the original.

Unlike copy number variations, sequence composition of satDNA monomers can remain remarkably stable over tens to hundreds of millions of years, with conserved satDNAs showing little species-specific divergence in phylogenetic analyses [27,33,88,121,151,152,153]. Although the cause of long-term conservation is not quite clear, it is often discussed in terms of slow evolution of sequences repeated in tandem and/or their involvement in functional interactions, as well as demographic changes [27,121,124,132,153,154].

## 5. SatDNA Evolution: Copy Number Variations and SatDNA Library

A major characteristic of every array of tandem repeats is extreme variability in copy numbers, which makes satDNAs an intrinsically unstable genome component [34,139,155,156,157]. Mechanisms of non-reciprocal DNA exchanges are invoked in the dynamic gain or loss of monomer repeats [10,18,19,44,84,99,100,124,128,131,133,158,159].

The array length, i.e., the number of monomers repeated in continuity, varies from hundreds of bp to hundreds of Mb [10,52]. For example, long-read sequencing combined with chromatin immunoprecipitation and sequencing (ChIP-seq) disclosed complex alterations in satDNA composition, array length and chromatin structure in mouse (peri)centromeric regions [160]. Variations in the number and size of satDNA arrays are associated with heterochromatin heteromorphism of homologous chromosomes in the Heteropteran insect *Holhymenia histrio* [161]. In humans, variations in satDNA content are a basis of individual chromosomal heteromorphism, with predicted impact on chromosome pairing and segregation in meiosis and on the expression of neighboring genes [162].

Copy number variations in satDNAs may be involved in the molecular differentiation of sex chromosomes, by amplification of short autosomal arrays in one species into long arrays distributed on sex chromosomes in other species [32,163,164,165]. Furthermore, differential amplification of different satDNA families may contribute to differentiation of sex chromosomes [30,31,32,86,104,122,144,163,165,166,167]. Specific accumulation of some satDNAs in the evolution of W sex chromosomes of fish species was proposed to be useful in sex identification in aquaculture [167,168].

The same satDNA may differ in abundancy and organizational pattern in a different species. Thus, while a particular satDNA may undergo substantial amplification at one or more loci in a given species, resulting in long arrays and a higher genomic proportion, it may remain unamplified in a related species, persisting as low-copy number satDNAs organized in short arrays. In this regard, part of the species-specificity of satDNA profiles can be explained by changes in copy numbers of satDNA families inherited from the common ancestor as a set. This set shared by a group of closely related species is called the satDNA library, proposed by Fry and Salser [85]. Library differences between species are quantitative, arising from independent and presumably random variations in the copy number of each satDNA within every genome [20,32,33,124,153,169,170,171]. Therefore, the amplified satDNAs in one species could have low copy number counterparts in the other species, and vice versa. For example, a relic satDNA in one species found as non-centromeric short arrays, may be organized as long arrays at centromeres in another [20,27].

The original library hypothesis does not clearly address the fact that satDNA families can disappear and a new one can emerge in a different species [14,26,33,150,172]. In fact, classical studies led to the belief that the satDNA library comprises a few families that are shared by a few related species. In contrast, comprehensive analyses of satellitomes have revealed that most species possess a far greater diversity of satDNAs than was anticipated just a few years ago (reviewed in [14]). The term satellitome is broader than that of the library, as it includes not only shared satDNAs but also those that are species-specific, regardless of whether these may be satDNAs that have arisen de novo or have been lost in the other species. Therefore, the number of shared satDNAs is roughly inversely proportional to the phylogenetic distance among the compared species, as observed in [20,23,27,33,154,172,173,174]. In addition, distribution and conservation of satDNAs in the library can be blurred because of links with TEs, introducing the possibility of horizontal transfer [14,33,88,150,152,175].

**Figure 1:**
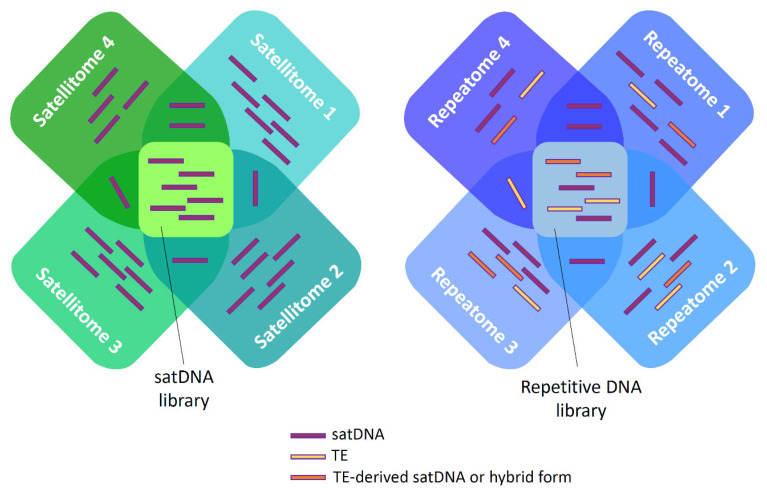
Scheme representing the differences between the satellite DNA library and satellitomes (**left**) and repetitive DNA library and repeatomes (**right**). The satellitomes and repeatomes shown (1–4) correspond to distinct species.

Based on the dominance of TE-related satDNAs in a group of oyster species, Tunjić-Cvitanić et al. [26] proposed the term “repetitive DNA library” which encompasses both the “TE library” and the “satDNA library”. The term “repetitive DNA library” should not be confused with the overall genomic repeat content, the repeatome (Figure 1). Obviously, a large number of “seed” sequences and diverse and complex mechanisms influence satDNA origin and evolution. In this regard, Balyayev et al. [150] introduced “library of mechanisms of origin” to include all features based on the common mechanisms of origin of shared satDNAs (reviewed in [14]).

With a plethora of tandem repeats of different repeat lengths, copy numbers and intra- and intergenomic distributions (explained in the following chapters), it would be reasonable to ask where to draw the line when defining a sequence as a satDNA? Independently of the variety of forms and circumstances that we observed within and between satellitomes, all examples of tandem repeats unequivocally represent satDNAs as it becomes evident that we are dealing with the same type of sequence at different stages of formation, depending on the processes involved in its origin, clustering and dissemination throughout the genome [10,14,19,50,60]. However, we recognize that the importance we place on the traditional concept of satDNA in our mental model may create doubts in this regard.

## 6. SatDNA Genomics: Satellitomes, SatDNA Abundancies and Genome Size

Each species possesses a characteristic satellitome, as revealed by novel NGS-based analyses, showing wide variation in satDNA diversity, from only a few families [27,110,176,177,178] to over a hundred; although, typically only a small number dominate in abundance [14,20,81,167,179,180]. Comparison of satellitomes in 63 plant and animal non-model species and in 60 species of *Drosophila* could not reveal any link between the number and abundancies of satDNA families within a species, nor could specific patterns be defined across the taxonomic groups (Tables 1 and 2 in [14], and references therein). In addition, neither the number nor overall abundance of satDNAs could be correlated with the genome size. A few illustrative examples are presented in Table 1.

Furthermore, it appears that there is no correlation between the number of satDNAs, their genome occupancy, and that of the most abundant satDNA. As a few examples, among 112 satDNAs composing 25% of the red palm weevil *Rhynchophorus ferrugineus*, the most abundant builds 20.4% [179], while in the larger genome of the fish *Megaleporinus elongatus* (0.78 Gb vs. 1.02 Gb, respectively), the most abundant of 140 satDNAs, builds only 0.48% [182]. The comparison between two bee species, *Melipona quadrifasciata* and *M. scutellaris* (0.26 Gb and 1.056 Gb, respectively) revealed 13 satDNA families in each species (11 shared) [184]. In *M. quadrifasciata* the 13 satellites compose 2.84% of the genome and the most abundant builds 0.95%, while 13 families compose 38.41% in the larger genome of *M. scutellaris*, with the most abundant satDNA building 38.2% [184]. In *Triatoma delpontei* ([171], Table 1)*,* the most abundant satDNA represents 18.16% of the genome (2.84 Gb and 51.1% of satDNA). In *Talpa aquitania*, with a genome size of 2.1 Gb, 16 different families, including telomeric sequences, represent 1.24% of the genome and the most abundant satDNA is 0.56% [185]. The most abundant satDNAs in two plant species with huge genomes, Killarney fern *Vandenboschia speciosa* [116] and wheat *Triticum aestivum* [22], of 10.5 and 16.95 Gb, respectively, represent 0.08% in the OJEN population of *V. speciosa* (11 satDNAs, 0.43% total) and 0.31% in *T. aestivum* (36 satDNAs, 2.53% total) (Table 1).

Also, it seems that there is no correlation between the total amount of all repetitive DNAs in the repeatome and satDNA content in the genome. For instance, despite a high repetitive DNA content of 76%, Killarney fern *Vandenboschia speciosa* contains only a small fraction of tandemly repeated DNA, about 0.4% [186]. Evolution of the large genome of this species (10.5 Gb) is therefore explained by expansion of TEs. Indeed, the differences in genome size of most species are generally attributed to varying concentrations of TEs and not to satDNA. However, there are a few examples in which these differences may be explained by variations in satDNA content [10,21,35,60,171,173,184,187,188,189].

Analyses of satellitomes across most species studied to date have revealed that a substantial fraction of sequences identified through genomic approaches correspond to low-copy number satDNAs, with some present in extremely low copy numbers. For instance, the *Talpa aquitania* satellitome comprises 15 satDNA families representing 1.24% of the genome, with 11 constituting together only 0.11% [185]. Among the 112 satDNAs identified in the red palm weevil *Rhynchophorus ferrugineus*, 102 make less than 0.1% [177]. In the ladybird *Hippodamia variegata*, 21 satDNAs are present at less than 0.1%, and 6 are below 0.01% [183]. Among the 165 identified satDNA families in the beetle *Chrysolina americana*, only three of them exhibit a remarkable amplification and accumulation [28].

Considering these low-copy percentages, even in large genomes they can represent only a few hundred bp which can be spread over several chromosomes. Short arrays may represent “seeds” for the formation of future long arrays [22,23,50,190]. Moreover, various types of such “seeds” (including TE-derived or TE-embedded tandem repeats) can exist as short arrays within the genome, contributing to the satellitome as species-specific low-copy tandem repetitive DNAs [10,19,20,26,50,94,180]. Alternatively, low-copy satDNAs can be the result of an extensive loss of formerly abundant repeats, such as in dynamic replacements of sequences in centromeric and pericentromeric regions [18].

As a conclusion, no correlation could be anticipated between the number, total abundance of satDNAs, the proportion of the most abundant satDNA(s), and genome size in closely related taxa—signifying the rapid and stochastic nature of changes and diverse evolutionary pathways in this class of genomic sequences and of the satellitome [14].

## 7. Clustered and Dispersed Patterns of SatDNAs

The paradigm that classical satDNAs are exclusively localized in heterochromatin has been disproven in recent years by rapid accumulation of results detecting satDNA sequences in euchromatic chromosomal segments, mostly in the form of short arrays (standalone or incorporated into TEs), as single monomers or their fragments (for example, [26,28,94,134,173,191,192,193,194]). Satellitome characterization, FISH mapping and/or in silico studies reveal two distinct organizational patterns of the genomic distribution of satDNAs.

The first organizational pattern reflects the long-standing view of satDNAs as clustered tandem arrays associated with heterochromatin [6,7,10,14]. Although satDNAs and their transcripts are known to regulate heterochromatin assembly and maintenance [10,60], some heterochromatin-associated satDNAs are also found in euchromatin. For example, highly abundant satDNA repeats clustered in (peri)centromeric regions of all chromosomes in *Tribolium castaneum* are dispersed throughout the euchromatin [195,196]. Similar dispersion of short arrays across the genome is observed for the migratory locust heterochromatic satDNA [50]. In the beetle *Chrisolina americana*, the three most abundant satDNAs form large domains in pericentromeric heterochromatin and their short arrays are interspersed in euchromatin together with the rest of the satellitome components [28]. Pericentromeric human alpha-satDNA is considered to be a source of copies detected in euchromatin, where they were spread probably via rolling-circle replication [197]. Similar as in the case of STRs, euchromatic copies of classical satDNAs can be positioned in the vicinity of genes and affect their expression [50,134,191,192,193,198,199,200].

The second pattern is opposite to the first. Tandem repeats are primarily placed within euchromatin, where arrays of monomers comparable to those of conventional satDNAs, are dispersed dominantly or even exclusively. Micro- and minisatellites can also be considered as forms of tandem repeats following this organizational pattern. Number and abundance of euchromatin-dominant satDNAs varies from species to species, up to the scenario where the entire satellitome is uncoupled from heterochromatin, as described in continuation.

An extreme case of the entire satellitome being composed of genome-through dispersed satDNAs was described in a species with monocentric chromosomes, the Pacific oyster *Crassostrea gigas* [94]. In this species heterochromatin is scarce, present only in the pericentromeres of one chromosome pair and subtelomeres of the other, that are, together with centromeres, populated mostly by TEs [201]. In fact, complete satellitomes (33–62 satDNAs) in several oyster species lack the compartmentalized satDNA organization regardless of the heterochromatin abundance and distribution in each of them. There, the euchromatic satDNA repeats are closely linked with the TEs of the Helitron superfamily, appearing as short arrays within TEs, and as standalone arrays, in fractions depending on the particular satDNA [26,94]. In addition to the euchromatic copies of monomers of the heterochromatin-dominant satDNA, the genome of the beetle *Tribolium castaneum* harbors relatively short arrays of low-abundant tandem repeats FISH-localized exclusively in euchromatin [202].

The euchromatin-dominant distribution is not an exclusive feature of minor satDNAs in the satellitomes. SatDNAs in the bug *Oxycarenus hyalinipennis* are dispersed in the euchromatic chromosomal regions of autosomes regardless of the abundance of each of them, while most of them are organized in long arrays constituting the heterochromatin of the Y chromosome [162]. In the hemipteran insect *Triatoma infestans*, out of 11 most abundant satDNAs, 7 were detected only in euchromatic chromosomal parts [173]. The three most abundant satDNAs of another hemipteran insect, *Rhodnius prolixus,* build together >3% of the genome and are distributed throughout the euchromatin of all chromosomes [203]. A recent study in two Chrysopini species also revealed that abundant satDNAs accumulated in euchromatin [194].

In the red palm weevil *Rhynchophorus ferrugineus*, the major satDNA (20.4% of the genome) predominates in euchromatin alongside several less abundant satDNAs, while also contributing to pericentromeric heterochromatin on all or some chromosomes, depending on the family [179]. In the ladybird beetle *Adalia bipunctata*, satellitome mapping shows dispersed organization in euchromatin, with a few satDNAs forming long heterochromatic arrays; in contrast, the same satDNAs in *A. decempunctata* are less abundant and only dispersed [25].

Although relatively few studies address this topic, dispersed distribution of satDNAs is suggested to be advantageous for plant and animal species with holocentric chromosomes. In plants of the genus *Rhynchospora*, 20–25 kb long arrays of the centromere-specific satDNA are dispersed along entire chromosomes in accordance with the distribution of the centromere-specific protein CenH3 [204,205]. Linked distribution of a specific satDNA and centromeric protein marker was described in the study of DNA sequences composing sites with the centromeric function in holocentric chromosomes of the root-knot nematode species [206]. These results may be comparable with the distribution of some satDNAs in holocentric Hemipteran insects mentioned above, although colocalization with centromere epigenetic markers was not explored in these studies [173,203]. In addition to the dispersed distribution of satDNAs in holocentrics, clustered organization can also be observed. Examples are several satDNAs of *Eleocharis* species in plants [207] and of *Mahanarva* species in insects [208].

It seems that a large scale of all possible distributional patterns of all forms of tandem repeats exists within and among species. Similar to what was found in the recent comparison of satellitomes by Šatović-Vukšić and Plohl [14], or in the most recent example of the satDNA library [33], it can be concluded that satellitome size and content, and distribution of satDNAs within the genome and among species, is extremely variable and is contingent on its nature. Further comparative satellitomics in every available species/species group might help to assess the whole range of diversity and some possible regularities.

## 8. Methodological Concerns, Risks Requiring Attention and Their Implications in SatDNA Research

In this section, we aim to briefly address the challenges associated with high-throughput methodologies for satDNA detection, while emphasizing current issues in the field. Novel approaches were shown to be of tremendous benefit for studying repetitive DNA content of non-model species without assembled genomes and have been used to define satellitomes and repeatomes in numerous (and constantly increasing) studies (reviewed in [14]). Nevertheless, several issues must be considered when employing these methodologies.

### 8.1. Where to Set the Threshold?

In some instances, the output of the program’s report of putative satDNA sequences in the genome is as low as 0.0001% (or even less). These are sometimes published as the official amount of newly detected satDNAs. However, if the genome and monomer size were considered and calculations were performed, this would represent the number of bp corresponding to only a few monomers on the genome level, and at times, not even a complete monomer. Given that short-read data do not provide the sequential order of the sequences, it becomes challenging to draw conclusions about the array length and tandem organization of these sequences. This raises the question of where to draw the line regarding the abundance thresholds for the sequences to be rightfully classified as satDNAs.

Additionally, it has been noted that satellitomes defined by the RepeatExplorer can vary significantly depending on the subset of reads analyzed. For instance, in four clustering analyses performed for each of the five oyster species, both the content and number of satDNAs differed notably [26]. In another study, the number of detected satDNAs in seven clustering for the insect species *Tribolium castaneum* ranged from 12 to 72 [190]. This underscores the need to conduct multiple clustering analyses with different sets of reads, followed by comparison and consolidation of the results, in certain (groups of) organisms. Of course, the threshold regarding the number of subsets included and the number of reads utilized must be assessed and optimized for each experimental system.

### 8.2. Index Hopping Poses a Serious Threat to the Analysis of Satellitomes

**Figure 2:**
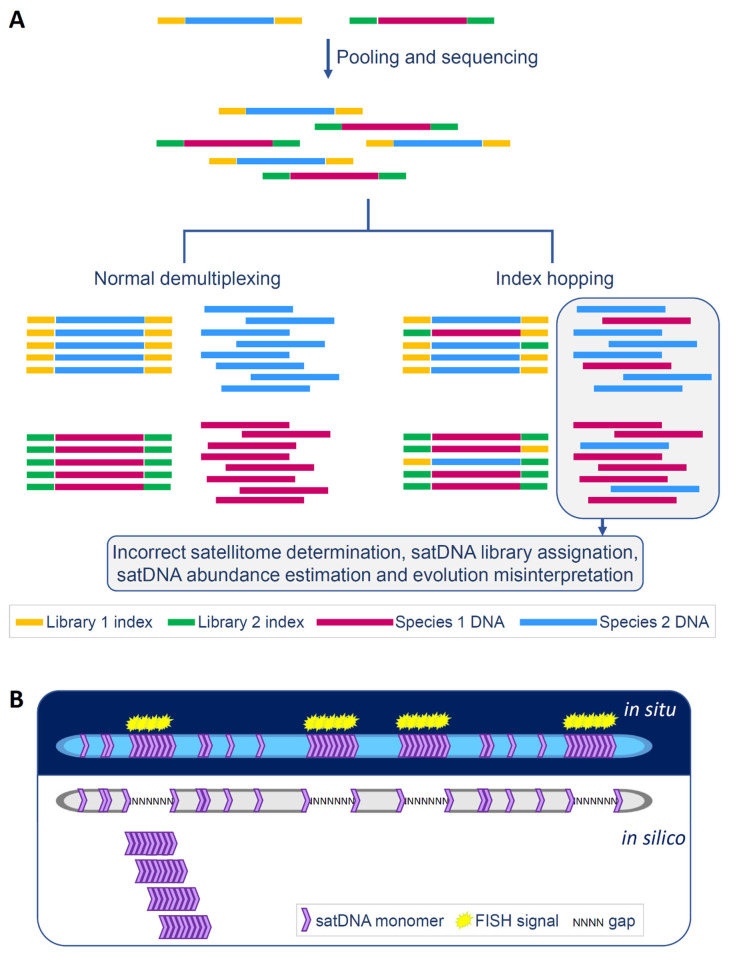
Index hopping and its potential consequences on satDNA studies (**A**). In situ and in silico localizations and how they complement each other (**B**).

Index hopping, also known as index swapping or barcode misassignment, can pose a significant challenge to the analysis of satellitomes. This phenomenon occurs when multiplexed samples are sequenced on NGS platforms like Illumina. Multiplexing allows for the addition of unique sequences, or indexes, to each DNA fragment during library preparation, enabling large numbers of libraries to be pooled and sequenced simultaneously in one sequencing run. However, it has been observed that a certain percentage of sequencing reads may be incorrectly assigned from one sample to another in the pool, due to index switching. Depending on the library preparation approach, the rate of index hopping has been reported to range from 0.3 to 3% [209]. This can lead to problems in data integrity (Figure 2A).

How does this affect satDNA research? If samples from two (or more) distantly related species are sequenced together, this can create misleading results, with reads from the most abundant satDNAs of one species being misattributed to the other. As a result, in the other species these reads may later be erroneously identified as low-abundance satDNAs. This would create a false impression of sequence conservation over long evolutionary periods. If the satDNA sequences are nearly identical in the sequencing outputs of the species, it could even lead to incorrect conclusions about their alternative evolutionary and distribution trajectories, such as horizontal transfer. Conversely, if samples from closely related species are sequenced together, this can also result in erroneous “members” in the library. Alternatively, specific reads may already be present in the related species. This overlap can complicate the estimated quantity of a certain satDNA in the genome, summing both “native” and introduced reads. Additionally, if the introduced reads differ from the native ones, this could lead to false interpretations about the presence of multiple subvariants of a certain satDNA within one genome. This may eventually lead to incorrect conclusions regarding the turnover and homogenization processes of satDNA sequences. In all cases, the technical bias caused by index hopping can result in misinterpretation of the obtained results. This point is difficult to control when the analyzed data are obtained from public databases. One potential solution to address this technical bias is the use of Unique Dual Index (UDI) and Unique Molecular Identifier (UMI) adapters. UMI sequencing assigns a unique sequence to each molecule in a sample prior to PCR amplification, while UDIs ensure that each sample index is specific to a given sample library. For example, these methods were utilized during the sequencing process for analyzing the satellitomes of bivalve species [26]. As long as index hopping poses a threat to accurate satDNA analysis and comparative satellitomics, methods that avoid or minimize it should become standard practice.

### 8.3. Same Sequence, Different Organizational Forms

As previously mentioned, the same sequence can coexist in different organizational forms within the genome or across different genomes. This variability often leads to many tandem repeats being misclassified or remaining unclassified altogether. This issue is particularly pronounced in comparative analyses of related species. Some sequences recognized and characterized as satDNA in one species’ genome may appear absent from the satellitome generated by RepeatExplorer for a related species. However, upon closer examination, these sequences may be present in other genomes but classified differently or located in the unclassified section. As illustrated in the work performed by Tunjić-Cvitanić et al. [26], to comprehensively study the distribution of a specific sequence across related species, a series of analyses may be required. This process may involve several individual clustering, comparative clustering using a consensus dataset that includes satellitome data for all species and the application of additional bioinformatics programs or approaches, all followed by thorough manual curation.

### 8.4. Advantages and Disadvantages of In Situ and In Silico Localizations and How They Complement Each Other

In the experimental verification of bioinformatic results, FISH on chromosomes and chromatin fibers is a valuable method for exploring the distribution of satDNA repeats. However, for effective detection, satDNA repeats must be sufficiently abundant and/or clustered. Very short arrays and interspersed monomers are likely to fall below the detection threshold of this method. The ability to study genomic distribution by annotating satDNAs in silico on improved genome assemblies is becoming increasingly significant. Despite advancements in sequencing methodologies, assemblies still remain generally smaller than the actual genome size, with gaps predominantly found in segments enriched with repetitive DNAs [37,38,40]. Due to challenges in accurately placing nearly identical repeats within long arrays, these sequences usually appear in unplaced scaffolds or may be entirely excluded from genome assemblies. In this context, short arrays and single interspersed monomers have a greater chance of being accurately positioned. Therefore, satDNA research can greatly benefit from complementing and juxtaposing in situ and in silico localizations, as their strengths and weaknesses in presenting the localization of long arrays and short satDNA segments are fundamentally opposite (Figure 2B).

## 9. Conclusions

The classical concept of satDNA encompasses highly abundant tandemly repeated sequences predominantly located in heterochromatin, which are readily visualized using FISH. Thanks to the novel approaches, the study of satellitomes has revealed also the existence of many families of tandem repeats of very diverse origin scattered throughout the genome as dispersed short arrays like “seeds” that can eventually amplify, generating longer arrays. Consequently, the same genome may simultaneously contain low-copy tandem repeat families organized in short arrays alongside more abundant ones organized either in long arrays (frequently heterochromatin blocks) and short arrays, or either solely in long or numerous short arrays. Furthermore, the organization of each satDNA may differ between species, being abundant and organized in long arrays in one species but appear only as low-copy short arrays in another. SatDNA families can originate from various sequences and different mechanisms in the genome, with a significant proportion deriving from other repeated sequences, particularly TEs. Taken together, the diversity of scenarios underlying origins of tandem repeats and the diversity of their arrangements in a genome would represent different stages of the same process. Given this background, one might ultimately ask what satDNA is. The most effective way to answer this question is perhaps the simplest, to define it as tandemly repeated DNA, based on its main organizational feature. However, to be more precise, we could extend the definition to include any non-coding repetitive DNA sequence ranging in size from a few bp to several thousand, organized in tandem arrays of variable lengths, either localized or scattered in the genome, and which may alter the expression of nearby genes and/or participate in the establishment of heterochromatin. Although this definition is not exhaustive and new insights continuously supplement and shape it, it still points to the unquestionable importance of these sequences for the architecture, functioning, and evolution of the genome.

## Figures and Tables

**Table 1:** Examples of relations between satellitomes and genome size.

Species	Genome Size (Gb)	satDNAs in the Satellitome	% of the Genome	Reference
Crayfish *Pontastacus leptodactylus*	18.7	258	27.5	[81]
Wheat *Triticum aestivum*	16.95	36	2.53	[22]
Killarney fern *Vandenboschia speciosa*	10.5	11	0.43	[116]
Plant *Chrysanthemum nankingense*	3.07	few microsatellites	0.16	[181]
Freshwater fish *Megaleporinus elongatus*	1.02	140	5	[182]
Red palm weevil *Rhynchophorus ferrugineus*	0.78	112	25	[179]
Pacific oyster *Crassostrea gigas*	0.6	52	6.3	[94]
Kissing bug *Triatoma delpontei*	2.84	160	51.1	[171]
Ladybird beetle *Hippodamia variegata*	0.28	29	14.7	[183]

## Data Availability

No new data were created or analyzed in this study. Data sharing is not applicable to this article.

## Abbreviations

The following abbreviations are used in this manuscript:

satDNAs	satellite DNAs
TEs	transposable elements
FISH	fluorescent in situ hybridization
